# Effects of Crude Rice Bran Oil and a Flaxseed Oil Blend in Young Horses Engaged in a Training Program

**DOI:** 10.3390/ani12213006

**Published:** 2022-11-02

**Authors:** Kayla C. Mowry, Timber L. Thomson-Parker, Cruz Morales, Kalley K. Fikes, Kyle J. Stutts, Jessica L. Leatherwood, Mark J. Anderson, Rachelle X. Smith, Jessica K. Suagee-Bedore

**Affiliations:** 1 School of Agricultural Sciences, College of Science and Engineering Technology, Sam Houston State University, Huntsville, TX 77340, USA; 2 Department of Animal Science, College of Agriculture and Life Sciences, Texas A&M University, College State, TX 77843, USA; 3 Sam Houston State University Analytical Laboratory, Sam Houston State University, Huntsville, TX 77341, USA

**Keywords:** creatine kinase, essential fatty acids, inflammation, interleukin-1β, lactic acid, muscle damage

## Abstract

**Simple Summary:**

Rice bran oil and flaxseed oil contain omega-3 fatty acids with the potential to reduce post-exercise inflammation and muscle damage. This study measures interleukin-1β and creatine kinase concentrations and fatty acid profiles in lightly worked, young horses undergoing a 16-min incremental exercise test after 60 days of oil consumption. Consuming crude rice bran oil or flaxseed oil for 60 days may benefit lightly worked, young horses by reducing training-program-related increases in interleukin-1β, while a flaxseed oil blend may reduce exercise-induced increases in creatine kinase. Additionally, the flaxseed oil blend has the potential to increase plasma omega-3 and omega-6 fatty acids.

**Abstract:**

Rice bran oil and flaxseed oil contain omega-3 fatty acids with the potential to reduce post-exercise inflammation and muscle damage. This study measures plasma interleukin-1β and creatine kinase and fatty acid profiles in lightly worked, young horses (*Equus caballus)* undergoing an exercise test after 60 days (d) of oil consumption, where the oil replaced 25% of concentrate calories. Treatments consisted of CON (no oil), FLAX (flaxseed oil blend), and RICE (crude rice bran oil). Blood was collected pre-exercise, and again at 1 min, 30 min, 24 h, 48 h, and 72 h post-IET. Data were analyzed by repeated measures ANOVA. Plasma creatine kinase activity was not different in CON during the study, greater (*p* < 0.05) in RICE from pre-exercise to 30 min post-exercise across all exercise tests, and lesser (*p* < 0.05) in FLAX at 30 min post-exercise on d 30 compared to d 0. Plasma interleukin-1β was greater (*p* < 0.01) in CON on d 60, but no differences were observed in FLAX and RICE throughout the study. Plasma alpha-linolenic and linoleic acids were greatest (*p* < 0.05) in FLAX after 30 d of inclusion, while CON horses had greater (*p* < 0.05) EPA across all exercise tests and DHA after 60 d. These results indicate that 60 d of inclusion of crude rice bran oil or a flaxseed oil blend may benefit lightly worked, young horses by reducing training-program-related increases in interleukin-1β, while a flaxseed oil blend may reduce exercise-induced increases in creatine kinase. Additionally, the flaxseed oil blend has the potential to increase plasma omega-3 and omega-6 fatty acids. Replacing 25% of concentrate calories with flaxseed or rice bran oil has potential benefits for young horses in training.

## 1. Introduction

Horses (*Equus caballus)* participate in a variety of sports, ranging from high intensity, short duration events that substantially elevate plasma lactate, such as thoroughbred racing [1], to lower-intensity sports that elevate lactate to a lesser degree, such as show jumping [2]. An increase in plasma lactate, an indicator of anaerobic metabolism, is associated with greater muscle soreness post-exercise [3], with muscle soreness further indicating some degree of muscular damage [4]. Muscle damage is an important component of muscle remodeling post-exercise, a mechanism that ultimately improves exercise capacity [5]. Both inflammation, which occurs in response to physical muscle damage, and anti-inflammation, which dampens the inflammatory response after cellular debris is cleared, are important phases of post-exercise muscle remodeling [5,6]. Muscle damage and inflammation may be measured in part through biomarkers such as creatine kinase (CK) and inflammatory cytokines, such as interleukin-1β (IL-1β) [7,8]. Reducing the inflammatory response after exercise appears to have the potential for increasing the ability of horses to perform repeated bouts of exercise [9], and the conditioning level appears to alter the blood peripheral mononuclear response to exercise, with fitter horses have lower production of inflammatory cytokines after exercise [10].

Oils such as rice bran and flaxseed are marketed as multipurpose equine nutritional supplements. Both oils contain components that reduce oxidative damage and inflammation, including phytosterols and vitamin E, which contain tocopherols and tocotrienol [11]. Both oils contain essential fatty acids; however, flaxseed oil contains a greater amount of the anti-inflammatory omega-3 fatty acid, alpha-linolenic acid (ALA), than rice bran oil. Generally, rice bran oil has a low omega-3 to 6 ratio (1:19), while flaxseed oil contributes more omega-3 fatty acids at 1:3 [12]. While there is no published ideal ratio for horses, it has been suggested that providing a diet with a greater omega-3 to 6 ratio can be more beneficial by helping to reduce inflammation and muscle damage post-exercise [13]. Additionally, rice bran oil contains γ-oryzanol, a mixture of antioxidant compounds that have previously been shown to lower blood lipids and oxidative stress [14,15]. In general, dietary fat supplementation increases caloric density as well as improves thermoregulation and reduces lactic acid concentrations during intense exercise in horses [16,17]. Adapting eventing [18] and endurance [19] horses to a high-fat diet has been shown to increase fatty acid oxidation, which, in turn, spares the utilization of muscle glycogen and blood glucose. Further, horse owners use fat supplements for improved coat conditions and the above-mentioned nutritional benefits [17].

The objective of this study is to determine the effects of replacing 25% of concentrate calories with either crude rice bran oil or a flaxseed oil blend of plasma concentrations of lactate, glucose, IL-1β, CK, and heart rates after intense exercise in young horses engaged in an introductory ground training program. Fatty acid profiles, body fat estimates, and muscle scores were also determined. It is hypothesized that horses consuming either oil would have reduced plasma lactate and IL-1β concentrations, reduced plasma CK activity, and increased plasma omega-3 fatty acids without loss in muscle mass or body fat gain.

## 2. Materials and Methods

### 2.1. Horses and Diets

**Figure 1:**
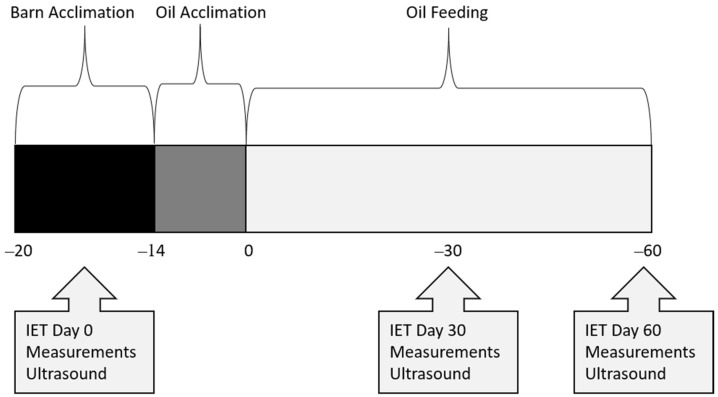
Timeline of data collection in young horses during a 60-day feed trial.

All procedures and animals used in this study were approved by the Sam Houston State University Institutional Animal Care and Use Committee (Protocol Number: 20-01-28-1042-3-01). Twelve healthy quarter horses were blocked by age into two groups: 18–23 months (n = 9) and 24–30 months (n = 3), then randomized within block into treatments of control (CON; n = 4), rice bran oil (RICE; n = 4), or a flaxseed oil blend (FLAX; n = 4) for a 60-day study. Horses were dewormed with ivermectin (Zimecterin, Boehringer Ingelheim, Duluth, GA, USA) 3 weeks (wk) before day (d) 0, then started a 1-week acclimation period to receiving a concentrate (SafeChoice Original, Cargill Animal Nutrition, St. Paul, MN, USA) at 0.7% of their BW daily, which approximated 40% of DE requirements. During acclimation, Coastal bermudagrass hay was fed at 1.5 to 2% BW daily. Following acclimation to the barn and concentrate feeding, BW was obtained, and diets adjusted to feed 60% of DE requirements from hay and 40% from concentrate (NRC, 2007). An acclimation to oil inclusion started 14 d before d 0 (Figure 1). Horses fed an oil treatment had 25% of their daily calorie requirement replaced with either crude rice bran oil (Riceland^®^, Stuttgart, AR, USA) or a flaxseed oil blend (Soybean oil, cold-pressed organic flaxseed oil; Animed™, Winchester, KY, USA; Table 1). The oil was measured, poured over their concentrate feed, and then mixed thoroughly to ensure adherence to the pellets. On average, daily DE intake was 21.58 Mcal for CON, 19.75 Mcal for FLAX, and 19.75 Mcal for RICE (Table 2). All horses were housed in 3.66 × 3.66 m stalls (Priefert, Mt. Pleasant, TX, USA) and were allowed 30 min of turnout time daily on a dry lot. Each horse also participated in a behavior and training class, which consisted of light groundwork 2 to 3 days a week, with no riding. Activities included lunging and round penning at walk, jog, and lope, saddled and unsaddled, as well as desensitizing to various objects. The full duration of exercise each day was approximately 30 min.

Measurements were obtained for body weight (BW), body condition score (BCS), cresty neck score (CNS), intramuscular fat (IMF), rump fat thickness (RFT), forearm and gaskin circumference, and longissimus muscle area (LMA) at 3 wk before d 0, and again on d 30 and 60 (Figure 1). Body weight was determined using a standard digital livestock platform scale. Body condition score was determined by using a 1–9 scale, where 1 = emaciated and 9 = obese [20]. Cresty neck scores were assigned using a 0–5 scale [21]. Several ultrasound measurements were recorded by a certified technician (Designer Genes Technologies, Inc., Harrison, AR, USA), including IMF (cm), RFT (cm), and LMA (cm^2^). Images were also evaluated by the same certified technician, who was blinded to dietary treatments. Forearm and gaskin circumference were measured in cm using a soft tape measure around the widest point. Measurements for BCS, CNS, and forearm and gaskin circumference were obtained by a single, trained individual who was blinded to dietary treatments.

### 2.2. Incremental Exercise Test

An IET was conducted on an automated horse walker (Priefert, Mt Pleasant, TX, USA) 3 wk before d 0, and again on d 30 and 60 of oil inclusion (Figure 1). All horses had free choice hay until the start of their exercise test to ensure similarities with fed states. The IET was conducted counterclockwise and consisted of 10 min at 16.1 kph, 2 min at 19.3 kph, 2 min at 22.5 kph, and 2 min at 25.7 kph or until exhaustion. Exhaustion was indicated when the horse struggled to keep pace with the automatic exerciser. Horses were then hand-walked for 30 min after completion of the exercise test. Blood samples were obtained via jugular venipuncture 4.5 h before each exercise test (fasting) and at 1 min, 30 min, 24 h, 48 h, and 72 h post-exercise. Whole blood was collected into evacuated tubes (Vacutainer, BD, Franklin Lakes, NJ, USA) coated with either lithium heparin (lipids, creatine kinase, IL-1β) or sodium fluoride/potassium oxalate (glucose and lactate) and stored on ice until centrifugation. Blood was then centrifuged for 10 min at 1500× *g*, and plasma was aliquoted and stored at −80 °C until further analysis. Heart rates were obtained via stethoscope, by experienced personnel, in the horse’s stall directly before the IET and 1 and 30 min post-IET on d 0 and 30. On d 60, heart rates were obtained via stethoscope in the horse’s stall directly before the IET and 30 min post-IET, with max heart rates obtained via heart rate monitor (KER Clockit Bluetooth Heart Rate Monitor, Polar Electro, Bethpage, NY, USA).

### 2.3. Sample Analysis

Plasma from collected blood was thawed at room temperature and analyzed to determine lactate, glucose, IL-1β concentrations, CK activity, and fatty acid percentages. Lactate and glucose concentrations were analyzed using a Yellow Springs Instruments (YSI) 2900 Biochemistry Analyzer (Xylem Analytics, Rye Brook, NY, USA).

Interleukin-1β concentrations were obtained using the Equine IL-1β ELISA Kit from Kingfisher Biotech Inc. (St. Paul, MN, USA) in duplicate and according to previously published methods (Suagee-Bedore et al., 2017). Plates were then read at 450 nm using a SpectraMax^®^190 plate reader (Molecular Devices, San Jose, CA, USA).

Creatine kinase activity was obtained using the EnzyChrom™ Creatine Kinase Assay Kit from BioAssay Systems (Hayward, CA, USA) according to the manufacturer’s provided protocol. The plate was covered and incubated at 37 °C inside a SpectraMax^®^190 plate reader (Molecular Devices, San Jose, CA, USA) and read at 340 nm at 20 and 40 min of incubation. The following equation was then used to calculate CK activity:

CK (U/L) = (OD_40min_ − OD_20min_/OD_Calibrator_ − OD_Water_) × 150 (1)

The profile of fatty acids in plasma was determined by the methylation of all lipids in plasma and quantifying peaks using gas chromatography to calculate the percent that each fatty acid contributed to total plasma lipids. Fatty acid percentages were determined using the protocol created by Perfield et al. [22] and modified by Corl et al. [23] and analyzed using gas chromatography (Agilent GC system 6890N, Agilent Technologies, Santa Clara, CA, USA). The obtained peaks were identified using specific markers (Pure Methyl Ester Standards 68D and 91, Nu-Check Prep Inc., Elysian, MN, USA), and converted to a percentage of total fatty acids. While chromatographic analysis isolated and quantified multiple fatty acids, data were only analyzed for the following: palmitic acid, oleic acid, linoleic acid, alpha-linolenic acid, eicosanoic acid, eicosatrienoic acid, eicosapentaenoic acid, and docosahexaenoic acid.

### 2.4. Feed and Oil Analysis

Composited samples for concentrate and hay were analyzed for nutrient and fatty acid concentrations, and samples for both oils were analyzed for fatty acid concentrations (Cumberland Valley Analytical Services, Waynesboro, PA, USA).

### 2.5. Statistical Analysis

All data collected were analyzed using the PROC MIXED procedure of SAS (SAS Enterprise Guide 7.1), with effects for d (0, 30, 60), treatment (CON, RICE, FLAX), time point (pre-exercise, 1 min post, 30 min post, 24 h post, 48 h post, 72 h post), and all interactions using repeated measures ANOVA for the time within d, with the horse as the subject. Data for lactate, glucose, IL-1β, and CK were log-transformed to meet normal standards for these parameters, then back-transformed as geometric means with a 95% confidence interval. One FLAX horse was removed from all statistical analyses of lactate, glucose, IL-1β, and CK due to injury before the d 60 IET. Differences between simple effects were determined using Tukey tests to reduce the potential for increased type-1 error rates.

## 3. Results

### 3.1. Diet Intake and Morphometrics

All horses readily consumed their respective diet (CON, RICE, or FLAX) throughout the duration of the 60-day feeding trial (Table 2). For body weight, there were no differences observed for treatment (*p* = 0.93; CON = 352.7 ± 21.7 kg, FLAX = 342.3 ± 21.7 kg, RICE = 352.3 ± 21.7 kg) or treatment by day (*p* = 0.77; data not shown). There was a main effect of day (*p* < 0.001), with lower BW on d 0 (333.6 ± 12.7 kg) than d 30 (357.1 ± 12.7 kg) and d 60 (356.6 ± 12.7 kg). There were no differences observed between d 30 and 60 (*p* = 0.98).

For BCS, no differences were observed for the treatment by day interaction (*p* = 0.88; data not shown). There was a main effect of treatment (*p* = 0.05), with a greater BCS in CON (5.8 ± 0.1) than FLAX (5.4 ± 0.1). There were no differences observed between CON and RICE (5.6 ± 0.1; *p* = 0.46) or between FLAX and RICE (*p* = 0.32). There was also a main effect of day (*p* = 0.05), with tendencies for a lower BCS on d 0 (5.3 ± 0.1) than d 30 (5.8 ± 0.1) and d 60 (5.7 ± 0.1). There were no differences observed between d 30 and 60 (*p* = 0.97). There was no effect of d (*p* = 0.61; d 0 = 1.5 ± 0.1; d 30 = 1.4 ± 0.1; d 60 = 1.6 ± 0.1) or treatment by day (*p* = 0.16; data not shown) for CNS. There was a tendency for effect of treatment (*p* = 0.06), with a greater CNS in RICE (1.8 ± 0.1) than FLAX (1.2 ± 0.1). However, there were no differences observed between CON (1.6 ± 0.1) and FLAX (*p* = 0.17) or between CON and RICE (*p* = 0.71).

For IMF, there was no effect of treatment (*p* = 0.76; CON = 11.5 ± 0.6 cm; FLAX = 12.0 ± 0.6 cm; RICE = 12.1 ± 0.6 cm) or treatment by day (*p* = 0.16; data not shown). Intramuscular fat had a main effect of day (*p* < 0.001), with measurements being smaller on d 0 (10.5 ± 0.4 cm) than d 30 (12.4 ± 0.4 cm) and d 60 (12.6 ± 0.4 cm). There were no differences observed between d 30 and 60 (*p* = 0.84). There was no effect of treatment (*p* = 0.38; CON = 0.6 ± 0.06 cm; FLAX = 0.5 ± 0.06 cm; RICE = 0.5 ± 0.06 cm) or treatment by day (*p* = 0.86) on rump fat thickness (RFT); data not shown). There was a main effect of day (*p* < 0.001) for RFT, with a significant increase in RFT from d 0 (0.3 ± 0.04 cm) to d 30 (0.5 ± 0.04 cm) and another significant increase from d 30 to 60 (0.8 ± 0.04 cm).

There was no effect of treatment (*p* = 0.81; CON = 46.6 ± 1.1 cm; FLAX = 45.8 ± 1.1 cm; RICE = 46.7 ± 1.1 cm) or treatment by day (*p* = 0.73; data not shown) on forearm circumference. A main effect of day (*p* < 0.001) was observed for forearm circumference, with average circumference being lower on d 0 (42.0 ± 0.8 cm) than d 30 (48.8 ± 0.8 cm) and d 60 (48.3 ± 0.8 cm). There were no differences observed between d 30 and 60 (*p* = 0.79). There was no effect of treatment (*p* = 0.82; CON = 41.4 ± 0.9 cm; FLAX = 42.0 ± 0.9 cm; RICE = 42.0 ± 0.9 cm) or treatment by day (*p* = 0.76; data not shown) on gaskin circumference. There was a main effect of day (*p* = 0.02) with average circumference being lower on d 0 (41.0 ± 0.6 cm) than d 30 (42.3 ± 0.6 cm) and d 60 (42.3 ± 0.6 cm). No differences were observed between d 30 and 60 (*p* = 0.99). There was no effect of treatment (*p* = 0.32; CON = 81.7 ± 4.0 cm^2^; FLAX = 73.3 ± 4.0 cm^2^; RICE = 80.6 ± 4.0 cm^2^) or treatment by day (*p* = 0.41; data not shown) on LMA. There was a main effect of day (*p* < 0.001) for LMA, with measurements being smaller on d 0 (72.0 ± 2.4 cm^2^) than d 30 (81.3 ± 2.4 cm^2^) and d 60 (82.3 ± 2.4 cm^2^). There were no differences observed between d 30 and 60 (*p* = 0.35).

### 3.2. Plasma Fatty Acids

Palmitic acid had no effect of treatment (*p* = 0.14; CON = 13.4 ± 0.4%; FLAX = 12.4 ± 0.4%; RICE = 12.6 ± 0.4%) or treatment by day (*p* = 0.14; Table 3). There was a main effect of day (*p* < 0.01), with percentages being greater on d 0 (13.4 ± 0.2%) than d 30 (12.6 ± 0.2%) and d 60 (12.5 ± 0.2%). There were no differences observed between d 30 and d 60 (*p* = 0.84).

Oleic acid had an interaction for treatment by day (*p* < 0.01), with CON having greater percentages on d 30 (28.6 ± 0.6%) than d 60 (25.4 ± 0.6%; Table 3). There were no differences observed between CON on d 0 (27.5 ± 0.6%) and CON on d 30 (*p* = 0.75) or between CON on d 0 and CON on d 60 (*p* = 0.21). FLAX on d 30 (28.2 ± 0.6%) had lower (*p* < 0.05) percentages than RICE on d 30 (31.4 ± 0.6%). There were no differences observed between CON (28.6 ± 0.6%) on d 30 and FLAX on d 30 (*p* = 0.99). There was a tendency for CON on d 30 to have lower percentages than RICE on d 30 (*p* = 0.07). Horses on RICE on d 30 (31.4 ± 0.6%) had greater (*p* < 0.01) percentages than RICE on d 0 (27.0 ± 0.6%) and RICE on d 60 (27.7 ± 0.6%). There were no differences observed between RICE on d 0 and RICE on d 60 (*p* = 0.99). The average percentage of oleic acid was 27.0 ± 0.4% on d 0, 29.4 ± 0.4% on d 30, and 27.0 ± 0.4% on d 60. The average percent of oleic acid for each treatment was 27.2 ± 0.5% for CON, 27.4 ± 0.5% for FLAX, and 28.7 ± 0.5% for RICE.

Linoleic acid (LA) had an interaction for treatment by day (*p* < 0.05; Table 3); however, there were no differences in simple effects. The average percentage of LA for each treatment was 48.9 ± 0.5% in CON, 49.7 ± 0.5% in FLAX, and 48.9 ± 0.5% in RICE. The average percentage of LA by day was 48.4 ± 0.4% on d 0, 49.0 ± 0.4% on d 30, and 50.0 ± 0.4% on d 60.

Alpha-linolenic acid (ALA) had an interaction for treatment by day (*p* = 0.02), with FLAX on d 30 (2.9 ± 0.3%) having greater percentages than CON on d 30 (1.8 ± 0.3%) and RICE on d 30 (1.5 ± 0.3%; Table 3). There were no differences observed between CON and RICE on d 30 (*p* = 0.95). The average percentage of ALA was 2.2 ± 0.1% on d 0, 2.1 ± 0.1% on d 30, and 1.9 ± 0.1% on d 60. The average percentage of ALA for each treatment was 2.0 ± 0.2% for CON, 2.5 ± 0.2% for FLAX, and 1.7 ± 0.2% for RICE.

Eicosanoic acid had no effect of treatment (*p* = 0.73; CON = 0.4 ± 0.1%; FLAX = 0.3 ± 0.1% in FLAX; RICE = 0.4 ± 0.1% in RICE) or treatment by day (*p* = 0.56; Table 3). There was a main effect of day (*p* < 0.001), with lower percentages on d 60 (0.06 ± 0.08%) than d 0 (0.6 ± 0.08%) and d 30 (0.5 ± 0.08%). There were no differences observed between d 0 and 30 (*p* = 0.56).

Eicosatrienoic acid had a tendency for an effect of treatment by day (*p* = 0.09), with greater percentages in CON on d 0 (3.1 ± 0.2%) than CON on d 30 (2.0 ± 0.2%) and CON on d 60 (2.1 ± 0.2%; Table 3). No differences were observed between CON on d 30 and CON on d 60 (*p* = 1.0). FLAX on d 0 (3.0 ± 0.2%) had greater percentages than FLAX on d 30 (1.4 ± 0.2%) and FLAX on d 60 (1.5 ± 0.2%). No differences were observed between FLAX on d 30 and FLAX on d 60 (*p* = 1.0). RICE on d 0 (3.3 ± 0.2%) had greater percentages than RICE on d 30 (1.7 ± 0.2%) and RICE on d 60 (1.4 ± 0.2%). No differences were observed between RICE on d 30 and RICE on d 60 (*p* = 0.85). There was an effect of day (*p* < 0.001), with greater percentages on d 0 (3.2 ± 0.1%) than d 30 (1.7 ± 0.1%) and d 60 (1.7 ± 0.1%). No differences were observed between d 30 and d 60 (*p* = 0.90). There was also a tendency for effect of treatment (*p* = 0.07), with a tendency for greater percentages in CON (2.4 ± 0.1%) than FLAX (2.0 ± 0.1%). No differences were observed between RICE (2.2 ± 0.1%) and CON (*p* = 0.28) or between RICE and FLAX (*p* = 0.54).

For eicosapentaenoic acid (EPA), there was no interaction for treatment by day (*p* = 0.90; Table 3). There was a tendency for an effect of day, with a tendency for greater percentages on d 0 (0.3 ± 0.04%) than d 30 (0.2 ± 0.04%; *p* = 0.06). There were no differences between d 60 (0.3 ± 0.04%) and d 0 (*p* = 0.65) or between d 30 and 60 (*p* = 0.27). There was also a tendency for an effect of treatment, with greater percentages in CON (0.3 ± 0.04%) than RICE (0.2 ± 0.04%; *p* = 0.06). There were no differences observed between FLAX (0.3 ± 0.04%) and RICE (*p* = 0.21) or between CON and FLAX (*p* = 0.69).

Docosahexaenoic acid (DHA) had an interaction for treatment by day, with greater percentages in CON on d 60 (1.4 ± 0.3%) than FLAX on d 60 (−0.2 ± 0.3%; *p* = 0.03; Table 3). No differences were observed between RICE (0.9 ± 0.3%) and FLAX on d 60 (*p* = 0.13) or between RICE and CON on d 60 (*p* = 0.96). There was also a tendency for greater percentages in FLAX on d 0 (1.0 ± 0.3%) than FLAX on d 60 (−0.2 ± 0.3%; *p* = 0.07), and a tendency for greater percentages in FLAX on d 30 (1.3 ± 0.3%) than FLAX on d 60 (*p* = 0.06). No differences were observed between FLAX on d 0 or 30 (*p* = 0.99). The average DHA percentage was 1.2 ± 0.1% for CON, 0.7 ± 0.1% for FLAX, and 1.0 ± 0.1% for RICE. The average percentage of DHA by day was 1.0 ± 0.2% on d 0, 1.2 ± 0.2% on d 30, and 0.7 ± 0.2% on d 60.

### 3.3. Plasma Lactic Acid

**Figure 2:**
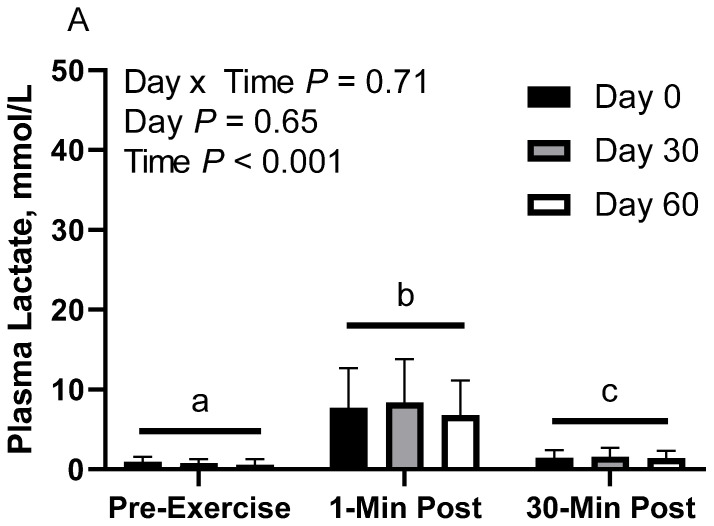

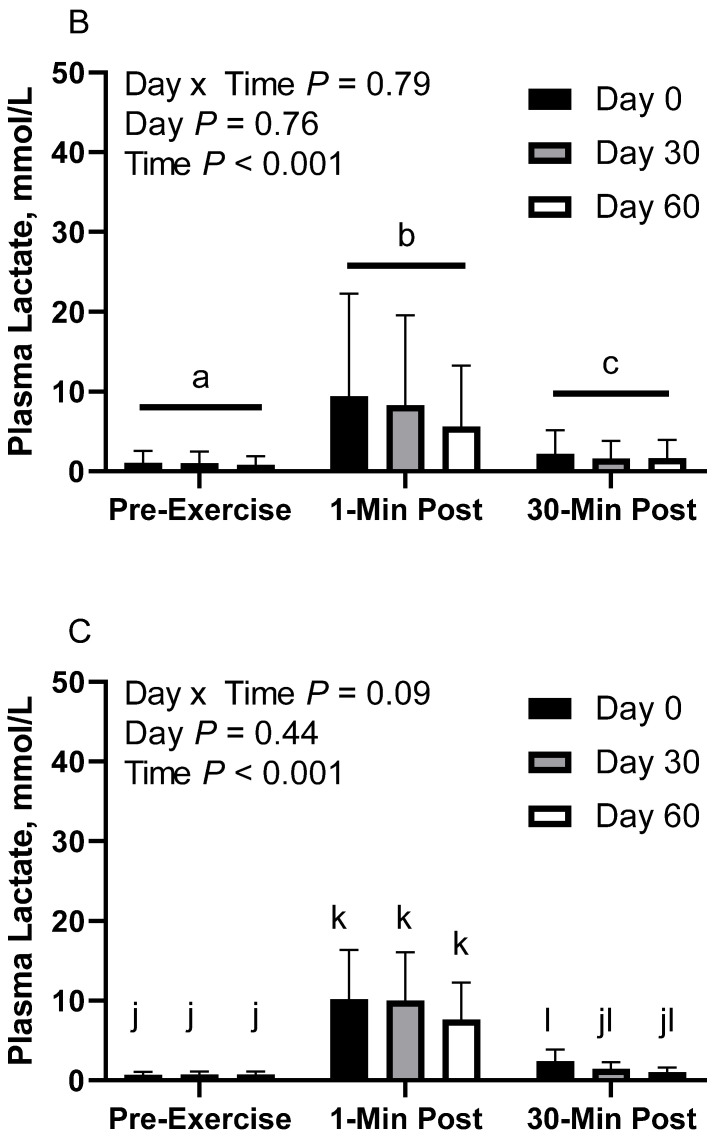
Geometric mean [LCI, UCI] fasting, 1 min post-exercise, and 30 min post-exercise plasma lactate concentrations in young horses fed 60% energy from hay (coastal bermudagrass) and 40% energy from concentrate (SafeChoice Original, Cargill Animal Nutrition, St. Paul, MN, USA) on d 0, 30, and 60 of a 60 d feeding trial. Where CON (**A**) did not receive dietary alteration, FLAX (**B**) had 25% of concentrate calories replaced with a flaxseed oil blend, and RICE (**C**) had 25% of concentrate calories replaced with crude rice bran oil. ^a,b,c^ Means not sharing a superscript differ *p* < 0.05. ^j,k,l^ Means not sharing a superscript differ *p* < 0.09.

In CON horses, there was no effect of day (*p* = 0.65) or day x time interaction (*p* = 0.70; Figure 2A). There was a main effect of time (*p* < 0.001), with greater geometric mean (95% CI) concentrations at 1 min post-exercise (7.6 [5.7, 10.1] mmol/L) than before exercise (0.7 [0.5, 1.0] mmol/L) and 30 min post-exercise (1.5 [1.1, 2.0] mmol/L), as well as 30 min post-exercise having greater concentrations than before exercise. Plasma lactate geometric mean concentrations were 2.2 [1.4, 3.3] mmol/L on d 0, 2.2 [1.4, 3.3] mmol/L on d 30, and 1.7 [1.1, 2.7] mmol/L on d 60.

In FLAX horses, there was no effect of day (*p* = 0.76) or day x time interaction (*p* = 0.79; Figure 2B). There was a main effect of time (*p* < 0.001), with greater geometric mean concentrations at 1 min post-exercise (7.6 [4.6, 12.5] mmol/L) than before exercise (1.0 [0.6, 1.6] mmol/L) and 30 min post-exercise (1.8 [1.1, 3.0] mmol/L), as well as 30 min post-exercise having greater concentrations than before exercise. Plasma lactate geometric mean concentrations were 2.8 [1.2, 6.4] mmol/L on d 0, 2.4 [1.0, 5.5] mmol/L on d 30, and 2.0 [0.9, 4.5] mmol/L on d 60.

In RICE horses, there was a tendency for a day x time interaction (*p* = 0.09), with greater geometric mean concentrations on d 0 at 1 min post-exercise (10.2 [6.4, 16.3] mmol/L) than before exercise (0.7 [0.4, 1.1] mmol/L) and 30 min post-exercise (2.4 [1.5, 3.9] mmol/L), as well as greater concentrations on d 0 at 30 min post-exercise than before exercise (Figure 2C). On d 30, 1 min post-exercise (10.0 [6.3, 16.1] mmol/L) had greater concentrations than before exercise (0.7 [0.4, 1.1] mmol/L) and 30 min post-exercise (1.4 [0.9, 2.3] mmol/L). There were no differences observed between before and 30 min post-exercise on d 30 (*p* = 0.12). On d 60, 1 min post-exercise (7.7 [4.8, 12.3] mmol/L) had greater concentrations than before exercise (0.7 [0.4, 1.2] mmol/L) and 30 min post-exercise (1.0 [0.6, 1.6] mmol/L). There were no differences observed between before exercise and 30 min post-exercise on d 60 (*p* = 0.83). There was also a main effect of time (*p* < 0.001), with greater geometric mean concentrations at 1 min post-exercise (9.2 [7.0, 12.1] mmol/L) than before exercise (0.7 [0.5, 0.9] mmol/L) and 30 min post-exercise (1.5 [1.2, 2.0] mmol/L), as well as 30 min post-exercise having greater concentrations than before exercise. There was no effect of day (*p* = 0.43). Plasma lactate geometric mean concentrations were 2.6 [1.7, 3.9] mmol/L on d 0, 2.2 [1.4, 3.3] mmol/L on d 30, and 1.8 [1.2, 2.7] mmol/L on d 60.

### 3.4. Plasma Glucose

**Figure 3:**
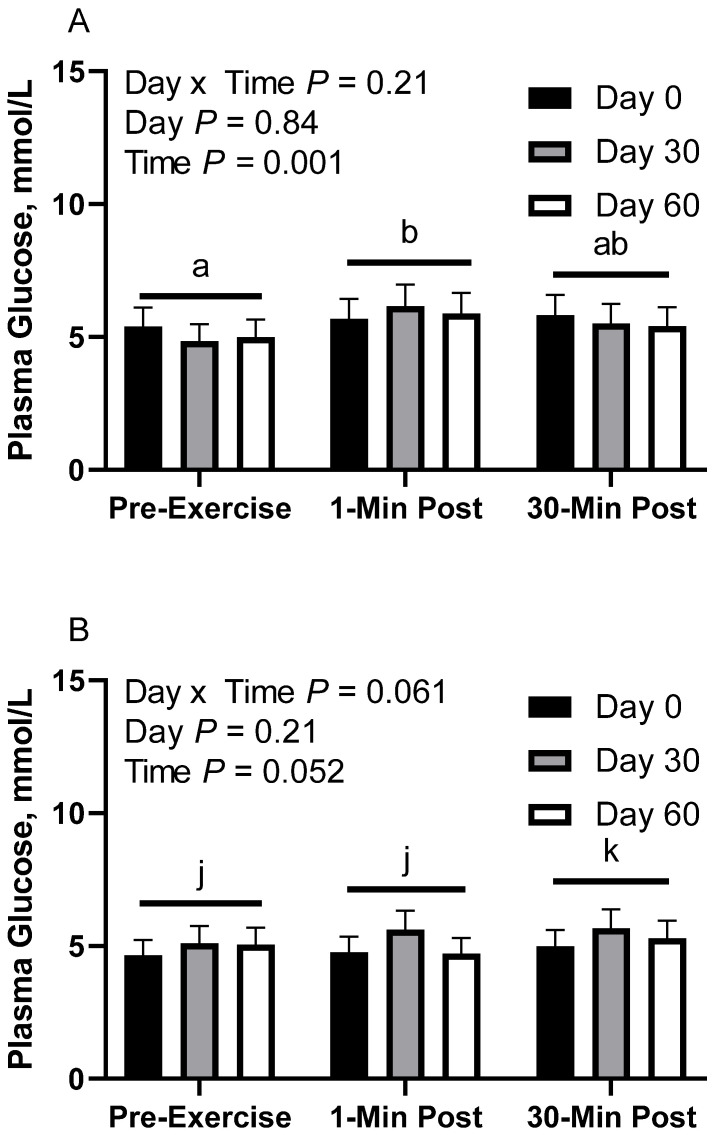

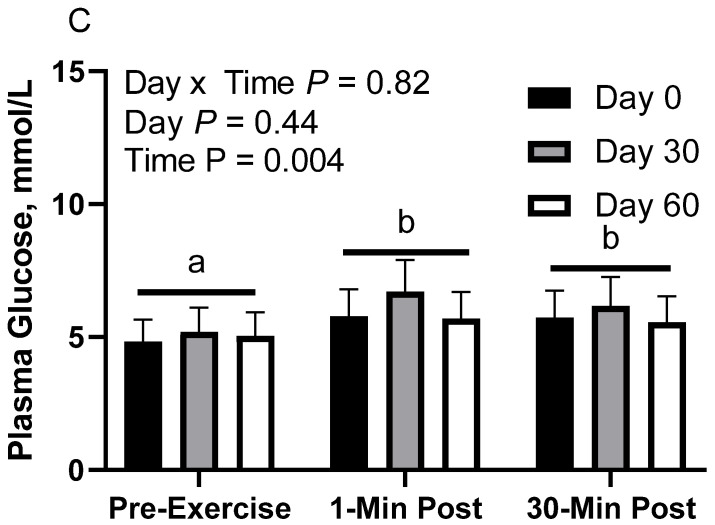
Geometric mean [LCI, UCI] fasting, 1 min post-exercise, and 30 min post-exercise plasma glucose concentrations in young horses fed 60% energy from hay (coastal bermudagrass) and 40% energy from concentrate (SafeChoice Original, Cargill Animal Nutrition, St. Paul, MN, USA) on d 0, 30, and 60 of a 60-day feeding trial. Where CON (**A**) did not receive dietary alteration, FLAX (**B**) had 25% of concentrate calories replaced with a flaxseed oil blend, and RICE (**C**) had 25% of concentrate calories replaced with crude rice bran oil. ^a,b^ Means not sharing a superscript differ *p* < 0.05. ^j,k^ Means not sharing a superscript differ *p* < 0.09.

In CON horses, there was no effect of day (*p* = 0.84) or day x time interaction (*p* = 0.21; Figure 3A) on glucose concentration. There was a main effect of time (*p* < 0.01), with greater geometric mean (95% CI) concentrations 1 min post-exercise (5.9 [5.5, 6.4] mmol/L) than before exercise (5.1 [4.7, 5.5] mmol/L). There was also a tendency for greater geometric mean concentrations 30 min post-exercise (5.6 [5.2, 6.0] mmol/L) than before exercise (*p* = 0.08). There were no differences observed between 1 and 30 min post-exercise (*p* = 0.24). Plasma glucose geometric mean concentrations were 5.6 [5.1, 6.2] mmol/L on d 0, 5.5 [5.0, 6.1] mmol/L on d 30, and 5.4 [4.9, 6.0] mmol/L on d 60.

In FLAX horses, there was a tendency for a day x time interaction (*p* = 0.06; Figure 3B); however, there were no simple effect differences. There was no effect of day (*p* = 0.20), but there was a main effect of time (*p* = 0.05), with a tendency for greater geometric mean concentrations 30 min post-exercise (5.3 [5.0, 5.7] mmol/L) than before exercise (4.9 [4.6, 5.3] mmol/L) and 1 min post-exercise (5.0 [4.7, 5.4] mmol/L). There were no differences observed between before exercise and 1 min post-exercise (*p* = 0.74). Plasma glucose geometric mean concentrations were 4.8 [4.3, 5.4] mmol/L on d 0, 5.5 [4.9, 6.1] mmol/L on d 30, and 5.0 [4.5, 5.6] mmol/L on d 60.

In RICE horses, there was no effect of day (*p* = 0.40) or day x time interaction (*p* = 0.82; Figure 3C). There was a main effect of time (*p* < 0.01), with lower geometric mean concentrations before exercise (5.0 [4.6, 5.5] mmol/L) than 1 min post-exercise (6.1 [5.5, 6.6] mmol/L) and 30 min post-exercise (5.8 [5.3, 6.4] mmol/L). There were no differences observed between 1 and 30 min post-exercise (*p* = 0.70). Plasma glucose geometric mean concentrations were 5.4 [4.8, 6.2] mmol/L on d 0, 6.0 [5.3, 6.8] mmol/L on d 30, and 5.4 [4.8, 6.2] mmol/L on d 60.

### 3.5. Heart Rates

On d 0, no differences were observed for heart rate (HR) before exercise (*p* = 0.92), 1 min post-exercise (*p* = 0.15), or 30 min post-exercise (*p* = 0.75; data not shown) between any of the treatments. Before exercise, HR was 46.0 ± 4.1 bpm for CON, 44.0 ± 4.1 bpm for FLAX, and 44.0 ± 4.1 bpm for RICE. At 1 min post-exercise, HR was 174.0 ± 4.3 bpm for CON, 165.0 ± 4.3 bpm for FLAX, and 161.0 ± 4.3 bpm for RICE. At 30 min post-exercise, HR was 62.0 ± 6.6 bpm for CON, 67.0 ± 6.6 bpm for FLAX, and 69.0 ± 6.6 bpm for RICE.

On d 30, no differences were observed for HR before exercise (*p* = 0.93), 1 min post-exercise (*p* = 0.32), or 30 min post-exercise (*p* = 0.94; data not shown) between any of the treatments. Before exercise, HR was 44.0 ± 2.2 bpm for CON, 44.0 ± 2.2 bpm for FLAX, and 45.0 ± 2.2 bpm for RICE. At 1 min post-exercise, HR was 155.0 ± 5.3 bpm for CON, 159.0 ± 5.3 bpm for FLAX, and 167.0 ± 5.3 bpm for RICE. At 30 min post-exercise, HR was 59.0 ± 4.9 bpm for CON, 61.0 ± 4.9 bpm for FLAX, and 59.0 ± 4.9 bpm for RICE.

On d 60, there were no differences observed for HR before exercise (*p* = 0.18), 30 min post-exercise (*p* = 0.88), or max HR (*p* = 0.34; data not shown) between any of the treatments. Before exercise, HR was 49.0 ± 2.9 bpm for CON, 40.0 ± 3.3 bpm for FLAX, and 44.0 ± 2.9 bpm for RICE. At 30 min post-exercise, HR was 57.0 ± 5.6 bpm for CON, 55.0 ± 6.4 bpm for FLAX, and 53.0 ± 5.6 bpm for RICE. Max HR was 215.0 ± 6.1 bpm for CON, 204.0 ± 7.1 bpm for FLAX, and 218.0 ± 6.1 bpm for RICE.

### 3.6. Plasma Interleukin-1β

**Figure 4:**
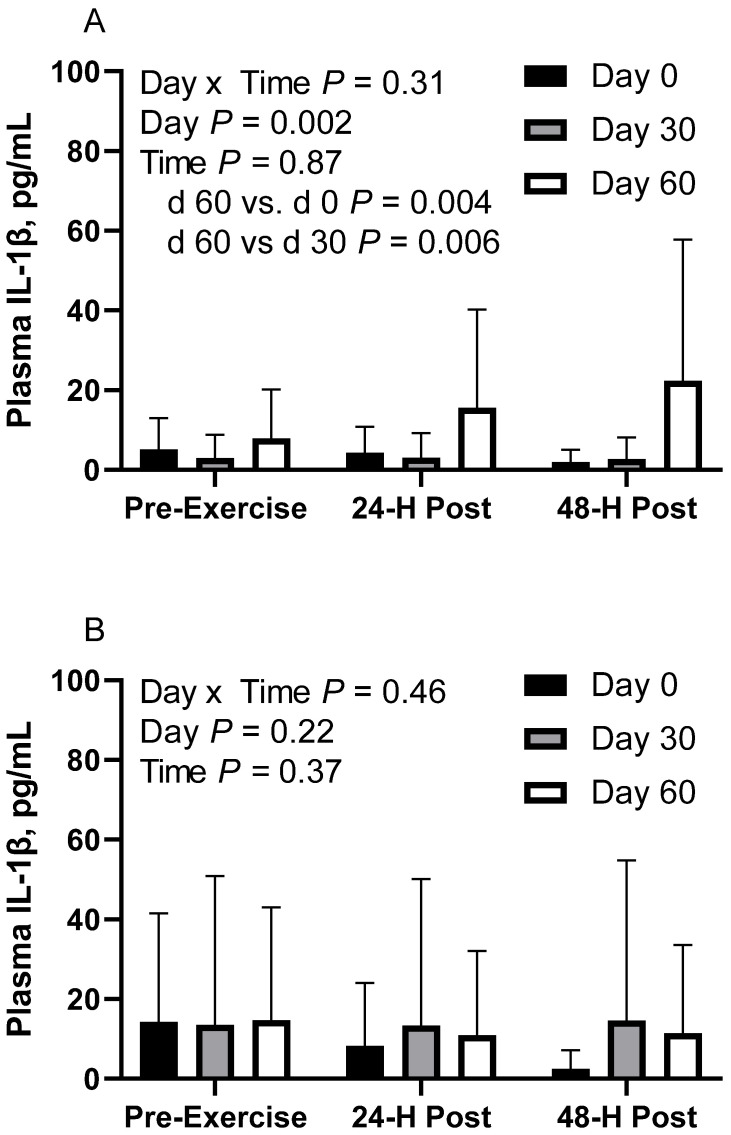

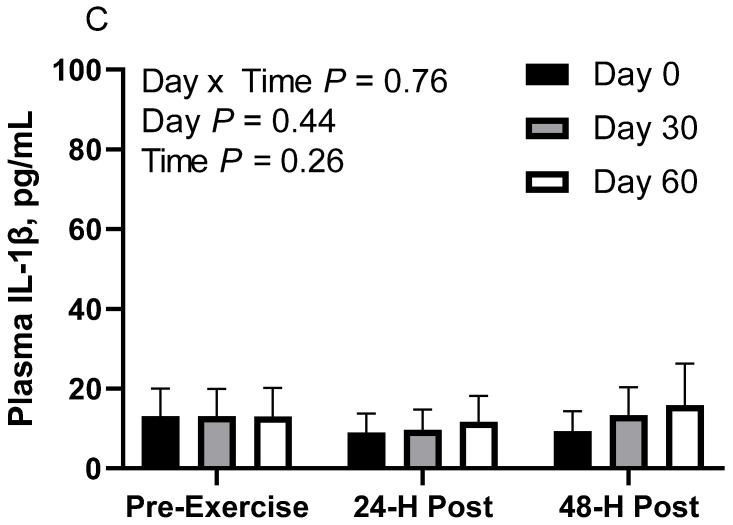
Geometric mean [LCI, UCI] fasting, 24 h post-exercise, and 48 h post-exercise plasma interleukin-1β concentrations in young horses fed 60% energy from hay (coastal bermudagrass) and 40% energy from concentrate (SafeChoice Original, Cargill Animal Nutrition, St. Paul, MN, USA) on d 0, 30, and 60 of a 60-day feeding trial. Where CON (**A**) did not receive dietary alteration, FLAX (**B**) had 25% of concentrate calories replaced with a flaxseed oil blend, and RICE (**C**) had 25% of concentrate calories replaced with crude rice bran oil.

In CON horses, there was no effect of time (*p* = 0.86) or day x time interaction (*p* = 0.31; Figure 4A) on plasma interleukin-1β (IL-1β). There was a main effect of day (*p* < 0.01), with greater geometric mean (95% CI) activity on d 60 (14.0 [7.9, 24.8] pg/mL) than d 0 (3.5 [2.1, 6.0] pg/mL; *p* = 0.004) and d 30 (2.9 [1.5, 5.7] pg/mL; *p* = 0.006). There were no differences observed between d 0 and 30 (*p* = 0.87). Plasma IL-1β geometric mean concentrations were 4.9 [2.8, 8.6] pg/mL before exercise, 5.9 [3.4, 10.3] pg/mL at 24 h post-exercise, and 4.9 [2.8, 8.7] pg/mL at 48 h post-exercise. One CON horse was removed from d 30 due to being a statistical outlier.

In FLAX horses, there was no effect of day (*p* = 0.21), time (*p* = 0.36) or day x time interaction (*p* = 0.46; Figure 4B). Plasma IL-1β geometric mean concentrations were 6.6 [3.6, 12.3] pg/mL on d 0, 13.8 [6.4, 30.2] pg/mL on d 30, and 12.3 [6.5, 23.0] pg/mL on d 60. For time, the concentrations were 14.2 [7.3, 27.6] pg/mL before exercise, 10.7 [5.5, 20.8] pg/mL at 24 h post-exercise, and 7.5 [3.8, 14.5] pg/mL at 48 h post-exercise. One FLAX horse was removed from d 30 due to being a statistical outlier.

In RICE horses, there was no effect of day (*p* = 0.43), time (*p* = 0.25), or day x time interaction (*p* = 0.76; Figure 4C). Plasma IL-1β geometric mean concentrations were 10.3 [8.0, 13.4] pg/mL on d 0, 11.9 [9.3, 15.3] pg/mL on d 30, and 13.4 [10.0, 18.0] pg/mL on d 60. For time, concentrations were 13.1 [10.3, 16.6] pg/mL before exercise, 10.1 [7.9, 12.8] pg/mL at 24 h post-exercise, and 12.6 [9.8, 16.2] pg/mL at 48 h post-exercise.

### 3.7. Plasma Creatine Kinase

**Figure 5:**
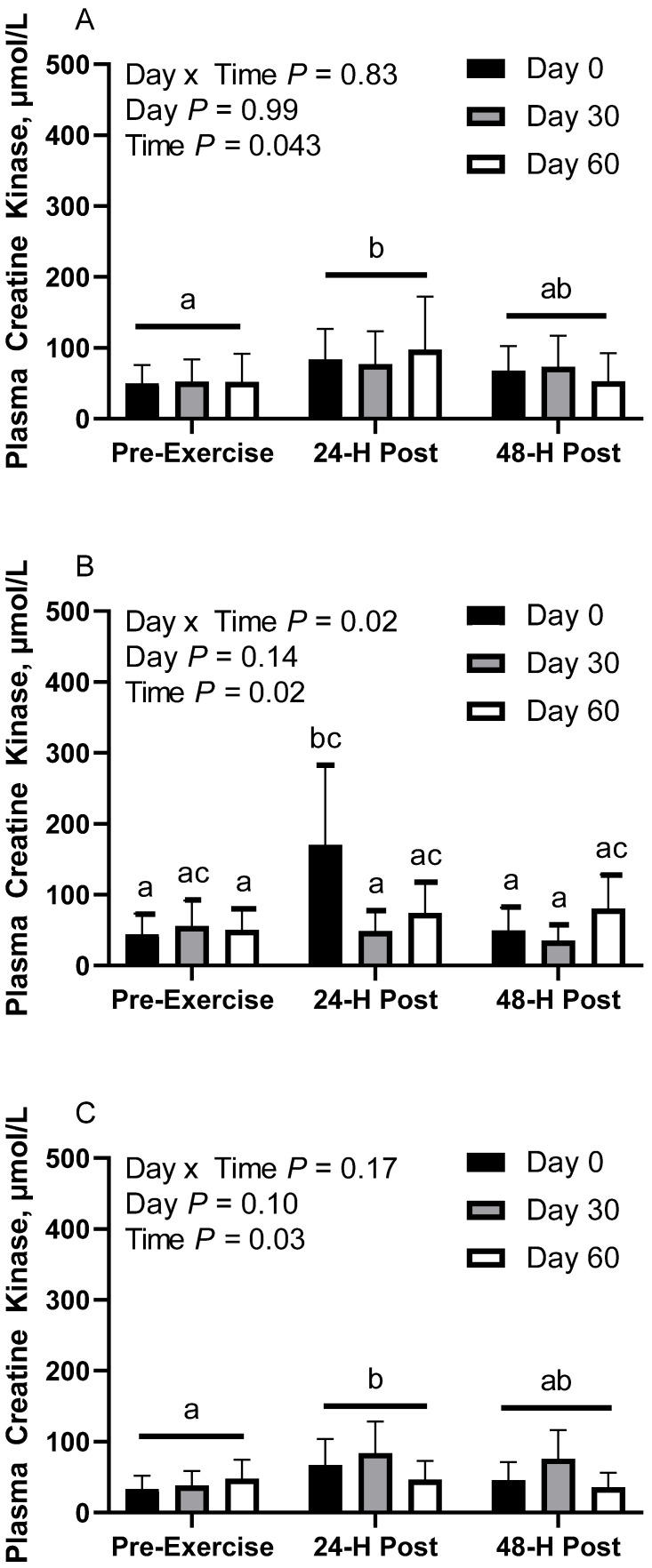
Geometric mean [LCI, UCI] fasting, 30 min post-exercise, and 24 h post-exercise plasma creatine kinase activity in young horses fed 60% energy from hay (coastal bermudagrass) and 40% energy from concentrate (SafeChoice Original, Cargill Animal Nutrition, St. Paul, MN, USA) on d 0, 30, and 60 of a 60-day feeding trial. Where CON (**A**) did not receive dietary alteration, FLAX (**B**) had 25% of concentrate calories replaced with a flaxseed oil blend, and RICE (**C**) had 25% of concentrate calories replaced with crude rice bran oil. ^a,b,c^ Means not sharing a superscript differ *p* < 0.05.

In CON horses, there was no effect of day (*p* = 0.98) or day x time interaction (*p* = 0.83; Figure 5A). There was a main effect of time (*p* = 0.04), with greater geometric mean (95% CI) activity at 30 min post-exercise (85.7 [64.9, 113.0] µmol/L) than before exercise (51.5 [39.0, 67.9] µmol/L). There were no differences observed between before exercise and 24 h post-exercise (63.8 [48.4, 84.2] µmol/L; *p* = 0.48) or between 30 min and 24 h post-exercise (*p* = 0.27). Plasma creatine kinase (CK) geometric mean activity was 65.7 [50.6, 85.2] µmol/L on d 0, 66.5 [49.9, 88.8] µmol/L on d 30, and 64.4 [46.1, 89.9] µmol/L on d 60. Two CON horses were removed from d 60 due to being statistical outliers, one of which was also removed from d 30.

In FLAX horses, there was an interaction for day x time (*p* = 0.01), with greater geometric mean activity on d 0 at 30 min post-exercise (170.8 [103.3, 282.1] µmol/L) than before exercise (44.5 [26.9, 73.5] µmol/L) and 24 h post-exercise (49.6 [30.0, 82.0] µmol/L; Figure 5B). There were no differences observed between before and 24 h post-exercise (*p* = 0.97). There was also a significant difference at 30 min post-exercise, with greater activity on d 0 (170.8 [103.3, 282.1] µmol/L) than d 30 (48.8 [29.7, 80.2] µmol/L). There were no differences observed at 30 min post-exercise between d 60 (74.2 [46.5, 118.3] µmol/L) and d 0 (*p* = 0.27) or at 30 min post-exercise between d 60 and d 30 (*p* = 0.91). Plasma CK geometric mean activity was 72.2 [52.1, 100.2] µmol/L on d 0, 45.9 [33.4, 63.2] µmol/L on d 30, and 67.1 [51.2, 87.9] µmol/L on d 60. Mean plasma CK activity for time was 50.1 [38.2, 65.6] µmol/L before exercise, 85.2 [65.1, 111.5] µmol/L at 30 min post-exercise, and 52.2 [39.8, 68.3] µmol/L at 24 h post-exercise.

In RICE horses, there was no effect of day (*p* = 0.10) or day x time interaction (*p* = 0.16; Figure 5C). There was a main effect of time (*p* = 0.02), with greater geometric mean activity at 30 min post-exercise (64.3 [50.3, 82.2] µmol/L) than before exercise (39.7 [31.0, 50.7] µmol/L). There were no differences observed between 24 h post-exercise (50.2 [39.3, 64.2] µmol/L) and before exercise (*p* = 0.35) or between 30 min and 24 h post-exercise (*p* = 0.32). Plasma creatine kinase geometric mean activity was 47.1 [36.1, 61.5] µmol/L on d 0, 62.6 [49.0, 80.1] µmol/L on d 30, and 43.4 [33.0, 57.1] µmol/L on d 60. This section may be divided by subheadings. It should provide a concise and precise description of the experimental results, their interpretation, as well as the experimental conclusions that can be drawn.

## 4. Discussion

The main objective of this study was to determine if 60 d of 25% calorie replacement with rice bran oil or a flaxseed oil blend would affect markers of metabolism, muscle breakdown, and inflammation post-exercise. Further objectives were to determine the effects of these oils on heart rate during exercise, plasma lipid profiles after replacement, body fat estimates, and muscling scores. It was hypothesized that crude rice bran oil, which contains omega-3 fatty acids, vitamin E, and γ-oryzanol, would lessen muscle damage and inflammation post-exercise in the oil-fed horses. The main findings of this study were that the exercise test induced anaerobic metabolism in all treatment groups, feeding a flaxseed oil blend increased linoleic acid (LA) and alpha-linolenic acid (ALA) percentages while decreasing eicosapentaenoic acid (EPA) and docosahexaenoic acid (DHA), and replacing 25% of concentrate calories with oil did not negatively impact muscle and fat parameters.

During the course of this study, BW increased and did not differ by treatment, which supports normal growth patterns in young horses receiving adequate nutrition. On average, horses gained approximately 0.3 kg/d, which is a suggested rate of daily gain for horses aged 18 months. While our horses ranged from 18 to 30 months, the majority of horses were closer to 18 months, a fact that should partly account for the average daily gain being more representative of younger horses [24]. It has also been shown that horses tend to be at 50% of their mature BW at 1 yr of age and at 75% of their mature BW at 2 yr of age [25]. Since long yearlings and 2-year-old horses were utilized, this could indicate that the horses in the current study had the potential to add approximately 25% of their mature BW during the 60 d. Additionally, BW comes from fat and muscle, along with other components not measured in the current study. Two body fat parameters, BCS and rump fat thickness (RFT), increased during the first 30 d in all treatments, which indicates that horses in all three treatments accrued body fat. Horses increased from 9.9% body fat to 12.3% body fat, which, according to previous studies, suggests that these horses increased from the low end to the high end of the normal body fat range, regardless of treatment [26,27]. Increases in both forearm and gaskin circumference were observed from d 0 to 30, indicating that the increase in BW from d 0 to 30 was not only due to the addition of fat but also included muscle accretion. This muscle accretion could have been due in part to the role of daily exercise. It is important to note that while replacing 25% of concentrate calories with oil removes a portion of crude protein and other nutrients supplied in concentrates, the muscle parameters did not differ in RICE or FLAX compared to CON. In summary, all horses increased in BW and body fat as well as added muscle, indicating that replacing concentrate calories with oil has no negative effects on fat and muscle parameters.

Flaxseed oil has been shown to consist of roughly 50% ALA, which makes it one of the richest sources of omega-3 fatty acids [28]. For the current study, the finding that plasma ALA percentages were greater in FLAX than in CON and RICE on d 30 may indicate that 30 d of flaxseed oil blend inclusion can help to increase ALA in plasma. Our results agree with those of Hansen et al. [29], in which horses fed flaxseed oil exhibited greater plasma ALA than control horses at 8 and 12 wk of oil inclusion. As a comparison, Hansen’s study added an additional 10% of flaxseed oil to the horse’s energy requirements, whereas in the current study, 10% of the total energy requirement came from a flaxseed oil blend. It is important to note that the flaxseed oil fed in Hansen’s study was 44% ALA, while the flaxseed oil blend in the current study was 9.2% ALA. The RICE horses consumed 58% of the amount of ALA consumed by FLAX horses, and it appears this was not a sufficient quantity to induce alterations to plasma content.

The increased ingestion of ALA did not correlate to increased plasma percentages of longer-chain omega-3 fatty acids. As shown in previous research, the conversion of ALA to EPA and DHA is limited due to competition with LA being converted to arachidonic acid [30]. Both of these fatty acids share the Δ6-desaturase enzyme, with ALA being the preferred substrate [30]. However, if LA is more prevalent in the diet, the metabolism of omega-6 fatty acids takes precedence over omega-3 fatty acid metabolism. All horses consumed more LA than ALA, which may have resulted in lower conversion rates of ALA to EPA and DHA [30]. Even after supplementation of ALA, studies have shown that there is little to no evidence of increases in circulating EPA and DHA in horses. However, in these studies, the horses consumed more ALA than LA [29,31,32]. For the current study, decreases in EPA after 30 d, as well as observations of CON horses having greater EPA and DHA than RICE and FLAX throughout the study, could also be explained by the greater amounts of LA in the FLAX and RICE treatments compared to CON, causing a decrease in the conversion rate of ALA to EPA and DHA. The increase in LA from d 0 to 30 is similar to the findings of Hansen et al. [29], in which plasma LA was greater in horses fed flaxseed oil than the control horses at 4, 8, and 12 wk of oil inclusion.

The incremental exercise test (IET) was shown to induce anaerobic exercise, as indicated by changes in plasma glucose, lactate, and heart rates. Lactate concentrations, as a measure of anaerobic threshold, are commonly used to assess the level of fitness in equine athletes [33,34]. In a study performed by Cabrera et al. [35], lactate concentrations reached the anaerobic threshold directly after high-intensity and maximum-intensity exercise and returned to aerobic levels during the recovery period, which was 10 min post-exercise. Therefore, the increase above 4 mmol/L in lactate concentrations at 1 min post-exercise across all treatments observed in the current study indicates that each horse was exercising anaerobically. Piccione et al. [33] also demonstrated a significant increase in lactic acid in show jumping horses as well as horses that participated in a 2 min treadmill running test. Both groups reached an anaerobic status, with lactate concentrations decreasing to baseline by 30 min post-exercise. For the current study, RICE lactate concentrations returned to baseline at 30 min post-exercise on d 30 and 60, with d 0 remaining higher. However, CON and FLAX remained elevated at 30 min post-exercise on d 0, 30, and 60. This could be explained by RICE containing γ-oryzanol, a mixture of ferulic acid esters that has been shown to decrease lactic acid concentrations after exercise [36].

Increases in plasma glucose concentrations for CON are consistent with a study from Ferraz et al. [37], where concentrations rose as the exercise intensity increased. Plasma glucose elevations towards the end of exercise have been related to the effects of catecholamines and glucagon on the liver, both increasing glucose release from the liver and decreasing the re-uptake of glucose by the liver [38], therefore increasing blood glucose concentrations. Some studies have indicated that fat supplementation improves glucose metabolism during exercise, which has further benefits on performance [39,40,41]. It is thought that fat supplementation drives an increase in glycogen stores or creates a glycogen-sparing effect by increasing fat utilization during exercise. These varied results may be due to a difference in fat type and amount, treatment duration in relation to metabolic adaptations, or a washout period being utilized [42]. During recovery, low free fatty acid concentrations may result in glucose being redirected for energy production. Therefore, providing a high-fat diet may spare muscle glycogen stores by increasing the availability of lipids during the recovery phase [43,44]. This could be used to explain why FLAX and RICE also had increased glucose concentrations post-exercise.

Interleukin-1β (IL-1β) is a pro-inflammatory cytokine that plays an important role in mediating inflammatory responses, and production is often stimulated by strenuous exercise [45]. In the current study, increases in post-exercise IL-1β concentrations after 30 d are similar to those seen in a study conducted by Fikes et al. [46], where IL-1β showed no changes in the unstimulated state but increased after a strenuous bout of exercise. These findings and those of the current study disagree with studies where conditioning decreased the post-exercise inflammatory response [10]; however, horses in the current study were not conditioned to perform the intense exercise of the IET. The horses used for the current study were lightly exercised 2–3 days per week for 30 min per session, with some of this time being spent on non-exercise tasks such as acclimation to wearing a saddle. This could indicate that the level of conditioning utilized prior to an exercise test is related to post-exercise inflammatory responses. In addition to the effect of conditioning, studies have suggested that the most effective option to inhibit the secretion of IL-1β is to inhibit the activity of caspase-1, the protease that produces mature IL-1β from its precursor protein [47]. Yan et al. [48] showed that in mice, omega-3 fatty acid supplementation helped to inhibit caspase-1 activity as well as IL-1β secretion by inhibiting NLRP3, a cytosolic protein complex responsible for activating caspase-1. Supplemental omega-3 fatty acids have also been shown to inhibit the production of IL-1β after an induced inflammatory response in humans [49] as well as in horses [50]. In the current study, FLAX and RICE showed no difference in IL-1β concentrations during the 60 d trial compared to CON, which experienced increases. This could be explained by both rice bran oil and the flaxseed oil blend containing high amounts of omega-3 fatty acids. More research is needed to further determine the effects of omega-3 fatty acid supplementation on IL-1β concentrations in exercising horses.

Creatine kinase (CK) concentrations greater than 10,000 U/L, which is equivalent to 167 µmol/L, are commonly used as an indicator of muscle damage in horses [51]. Concentrations typically rise after strenuous exercise and return to baseline by 24 h post-exercise, with a lesser rise in plasma CK indicating less muscle damage [52]. For the current study, in FLAX before calorie replacement with oil, the increase in CK activity post-exercise, followed by the decrease, corresponds to a study conducted by [53], where CK activity increased directly after exercise, then dropped down to pre-exercise values by 24 h post-exercise. Observations of lower CK activity for FLAX at 30 min post-exercise after feeding 30 d of flax oil could be explained by greater amounts of ALA present in the flaxseed oil blend. In agreement, other studies have shown that CK activity is markedly lower if horses are provided a high-fat diet through the use of top-dressed flaxseed or soy-based oils that contain high amounts of omega-3 fatty acids, such as ALA [54]. This finding is potentially due to the protective effects of omega-3 fatty acids on cell membranes [55]. This could explain why CK activity remained high at 24 h post-exercise for RICE and CON, as horses on these treatments consumed lower amounts of ALA than FLAX.

## 5. Conclusions

In summary, 30 to 60 d of inclusion of crude rice bran oil or a flaxseed oil blend may benefit lightly worked, young horses by reducing training-program-related increases in interleukin-1β, while only the flaxseed oil blend may help to reduce exercise-induced increases in creatine kinase. Results also indicate that neither oil induces a loss of muscle mass or an increase in body fat. Additionally, the flaxseed oil blend has the potential to increase plasma omega-3 and omega-6 fatty acids. Future research could potentially determine the effects of a blended combination of these oils and see how it compares in young growing horses vs. mature performance horses.

## Figures and Tables

**Table 1:** Nutrient composition of feeds and oils fed to young horses during a 60-day feeding trial.

Nutrient, DM Basis ^1^	Hay	Concentrate ^2^	RICE ^3^	FLAX ^4^
DE, MCal/kg	1.83	3.08	8.8	8.8
CP, %	10.9	16.3	-	-
ADF, %	36.3	16.7	-	-
NDF, %	52.1	31.2	-	-
EE, %	1.39	6.60	-	-
Ash, %	7.28	1.40	-	-
FA, % of Total EE				
C18:2 n6 (LA)	14.3	50.1	38.4	51.5
C18:3 n3 (ALA)	13.4	4.75	1.47	9.21
C20:5 n3 (EPA)	0	0	0	0
C22:6 n3 (DHA)	1.70	0	0	0
n3:n6 ratio	1.1	0.1	0.04	0.18
Calcium, %	0.41	1.92	-	-
Phosphorous, %	0.21	1.17	-	-
Magnesium, %	0.23	0.47	-	-
Potassium, %	1.72	1.27	-	-

^1^ Dry matter (DM); digestible energy (DE); crude protein (CP); acid detergent fiber (ADF); neutral detergent fiber (NDF); ether extract (EE), fatty acid (FA); linoleic acid (LA); alpha-linolenic acid (ALA); eicosapentaenoic acid (EPA); docosahexaenoic acid (DHA). ^2^ Cargill Animal Nutrition, St. Paul, MN. ^3^ Crude Rice Bran Oil, Riceland Foods^®^, Stuttgart, AR. ^4^ Flaxseed Oil Blend, AniMed™, Winchester, KY, USA.

**Table 2:** Average daily nutrient intake of young horses consuming hay and a pelleted concentrate with no oil (CON) or either crude rice bran oil (RICE) or a flaxseed oil blend (FLAX) replacing 25% of concentrate calories daily for 60 d.

	Treatment		
Nutrient	CON	RICE	FLAX
DE, Mcal	21.58	19.75	19.75
CP, g	1180.93	999.43	999.43
ADF, kg	2.93	2.73	2.73
NDF, kg	5.53	5.13	5.13
EE, g	275.13	400.28	400.28
18:2 n6, g (LA)	101.5	149.25	174.25
18:3 n3, g (ALA)	20.75	19.75	35.75
20:5 n3, g (EPA)	0	0	0
22:6 n3, g (DHA)	1.88	1.88	1.88
Calcium, g	82.92	61.48	61.48
Phosphorous, g	47.41	34.35	34.35
Magnesium, g	27.85	22.66	22.66
Potassium, g	152.93	138.78	138.78

**Table 3:** Mean ± SEM fatty acid profile of plasma lipids ^1^ in long yearlings consuming hay and a pelleted concentrate with no oil (CON) or either crude rice bran oil (RICE) or a flaxseed oil blend (FLAX) replacing 25% of concentrate calories daily for 60 d.

Treatment (TRT)		Plasma Fatty Acid, %				*p*-Value		
	TRT Mean	D 0	D 30	D 60	SEM	TRT	Day	TRT X D
16:0 Day Mean	---	13.4 ^a^	12.6 ^b^	12.5 ^b^	0.2			
CON	13.4 ± 0.4	13.7	13.4	13.3	0.4	0.14	0.004	0.15
RICE	12.6 ± 0.4	12.9	12.5	12.5	0.4			
FLAX	12.4 ± 0.4	13.5	12.0	11.7	0.4			
18: 1 Day Mean	---	27.0	29.4	27.0	0.4			
CON	27.19 ± 0.46	27.5 ^ab^	28.6 ^ad^	25.4 ^b^	0.6	0.08	0.001	0.002
RICE	28.73 ± 0.46	27.0 ^ab^	31.4 ^cd^	27.7 ^ab^	0.6			
FLAX	27.44 ± 0.46	26.3 ^ab^	28.2 ^ab^	27.8 ^ab^	0.6			
18:2 Day Mean	---	48.4	49.0	50.0	0.4			
CON	48.87 ± 0.46	48.0	48.3	50.3	0.7	0.41	0.03	0.04
RICE	48.86 ± 0.46	49.1	47.9	49.7	0.7			
FLAX	49.65 ± 0.46	48.1	50.8	50.0	0.7			
18:3 Day Mean	---	2.2	2.1	1.9	0.1			
CON	2.00 ± 0.15	2.2 ^abc^	1.9 ^ac^	1.9 ^abc^	0.2	0.007	0.24	0.03
RICE	1.68 ± 0.15	2.0 ^abc^	1.5 ^a^	1.52 ^ac^	0.2			
FLAX	2.51 ± 0.15	2.4 ^abc^	2.9 ^b^	2.3 ^abc^	0.2			
20:1 Day Mean	---	0.6 ^a^	0.5 ^a^	0.1 ^b^	0.1			
CON	0.43 ± 0.09	0.6	0.6	0.2	0.1	0.73	0.001	0.57
RICE	0.39 ± 0.09	0.6	0.6	0.0	0.1			
FLAX	0.33 ± 0.09	0.6	0.4	0.0	0.1			
20:3 Day Mean	---	3.2 ^a^	1.7 ^b^	1.7 ^b^	0.1			
CON	2.42 ± 0.12	3.1	2.0	2.1	0.2	0.07	0.001	0.09
RICE	2.15 ± 0.12	3.3	1.7	1.4	0.2			
FLAX	1.96 ± 0.12	3.0	1.4	1.5	0.2			
20:5 Day Mean	---	0.3 ^j^	0.2 ^k^	0.3 ^jk^	0.04			
CON	0.30 ± 0.04 ^j^	0.3	0.2	0.3	0.1	0.06	0.07	0.86
RICE	0.15 ± 0.04 ^k^	0.2	0.1	0.1	0.1			
FLAX	0.15 ± 0.04 ^jk^	0.4	0.1	0.3	0.1			
22:6 Day Mean	---	1.0	1.2	0.7	0.2			
CON	1.19 ± 0.12	1.0 ^ac^	1.2 ^a^	1.4 ^a^	0.3	0.04	0.20	0.03
RICE	0.96 ± 0.12	1.0 ^ae^	1.0 ^ae^	0.9 ^ae^	0.3			
FLAX	0.69 ± 0.12	1.0 ^ae^	1.3 ^ae^	−0.1 ^bcde^	0.3			

^1 ^Fatty acids presented as a percentage of total lipids. ^abcde^ Means with unlike superscripts differ *p* < 0.05. ^jk^ Means with unlike superscripts differ *p* < 0.1.

## Data Availability

Not applicable.
